# KDEON WK-11: A short antipseudomonal peptide with promising potential

**DOI:** 10.3389/fchem.2022.1000765

**Published:** 2022-11-17

**Authors:** Bruno Casciaro, Maria Rosa Loffredo, Floriana Cappiello, Niamh O’Sullivan, Carola Tortora, Rizwan Manzer, Sougata Karmakar, Alan Haskell, Syed K. Hasan, Maria Luisa Mangoni

**Affiliations:** ^1^ Laboratory Affiliated to Pasteur Italia-Fondazione Cenci Bolognetti, Department of Biochemical Sciences A. Rossi Fanelli, Sapienza University of Rome, Rome, Italy; ^2^ Department of Chemistry and Technology of Drugs, “Department of Excellence 2018–2022”, Sapienza University of Rome, Rome, Italy; ^3^ Iuventis Technologies Inc. (DBA Immunotrex Biologics), Lowell, MA, United States

**Keywords:** antimicrobial peptides, lipopolysaccharides, *Pseudomonas aeruginosa*, tryptophan, antibiofilm activity

## Abstract

The plight of antimicrobial resistance continues to limit the availability of antibiotic treatment effective in combating resistant bacterial infections. Despite efforts made to rectify this issue and minimise its effects on both patients and the wider community, progress in this area remains minimal. Here, we *de-novo* designed a peptide named KDEON WK-11, building on previous work establishing effective residues and structures active in distinguished antimicrobial peptides such as lactoferrin. We assessed its antimicrobial activity against an array of bacterial strains and identified its most potent effect, against *Pseudomonas aeruginosa* with an MIC value of 3.12 μM, lower than its counterparts developed with similar residues and chain lengths. We then determined its anti-biofilm properties, potential mechanism of action and *in vitro* cytotoxicity. We identified that KDEON WK-11 had a broad range of antimicrobial activity and specific capabilities to fight *Pseudomonas aeruginosa* with low *in vitro* cytotoxicity and promising potential to express anti-lipopolysaccharide qualities, which could be exploited to expand its properties into an anti-sepsis agent.

## 1 Introduction

Multidrug resistant (MDR) infections are now considered a serious threat that has ushered our society into a post-antibiotic era, putting at risk not only human health, but also the ecosystem and primary sectors of the economy. Infections caused by resistant Gram-negative bacteria (e.g., *Pseudomonas aeruginosa*) are difficult to treat and are associated with high morbidity and mortality rates (Kaye and Pogue, 2015). *Pseudomonas* can easily colonise human tissues, such as wounds or the lungs, producing biofilms and can provoke sepsis and septic shock due to the release of endotoxins. Antimicrobial resistance in this class of pathogenic bacteria is one of the most pressing challenges in the field of infectious diseases and the reduced number of new antibiotics on the market pushes research into new compounds with antimicrobial activity and alternative mechanism(s) of action (Doi et al., 2017). Antimicrobial peptides (AMPs) of innate immunity represent an interesting class of molecules with expanding properties and the potential for the development of new antibiotics (Mangoni et al., 2016; Casciaro et al., 2020; Rivas-Santiago et al., 2021; Zhang et al., 2021). These short cationic peptides interact with negatively charged components of the bacterial cell wall (lipoteichoic acid in Gram-positives and lipopolysaccharides, LPS, in Gram-negatives) and destabilize the integrity of the microbial membrane as a primary bactericidal mechanism of action. LPS molecules usually consist of three portions: 1) a hydrophobic moiety for anchoring LPS to the outer membrane, named lipid A; 2) an oligosaccharide core that contributes to maintaining outer membrane integrity; 3) and repeating oligosaccharide units in direct contact with the external milieu, named O Antigen (Hellman and Warren, 1999; Whitfield and Trent, 2014). Considering that the release of LPS is one of the major causes of endotoxin-induced production of inflammatory cytokines and septic shock (Morrison and Ryan, 1987), AMPs capable of killing pathogenic Gram-negative bacteria and neutralizing LPS, are in high demand. Lactoferrin is a perfect example; this is an iron-binding glycoprotein which is found in most exocrine secretions (Sinha et al., 2013), and provides a rich source of cationic and hydrophobic AMPs released upon proteolysis (Farnaud and Evans, 2003; Lizzi et al., 2009) that neutralize the effect of LPS-induced toxicity by binding to LPS molecules (Miyazawa et al., 1991; Appelmelk et al., 1994). Considering that the primary sequences of these lactoferrin-derived AMPs are rich in tryptophan, phenylalanine, and lysine residues, we designed a short AMP named KDEON WK-11 with two of these three residues consistently repeated in its primary structure (i.e., WWKKWWKKWWK). KDEON WK-11 was firstly analysed for its antimicrobial and antibiofilm activity, its mechanism of action followed by its structural conformation and cytotoxicity analysis.

## 2 Materials and methods

### 2.1 Peptide

KDEON WK-11 was purchased from IDT Inc (Integrated DNA Technologies Incorporated). KDEON WK-11 was synthesized by stepwise solid-phase F-moc synthesis with free ends at the N and C-terminals and a purity greater than 95%. High performance liquid chromatography (HPLC) spectrum of the peptide is reported in Supplementary Figure S1. The peptide was solubilized in PBS at a concentration of 2 mM. Electrospray ionisation mass spectrometry was performed using Diphosphoryl Lipid-A (Sigma Aldrich), chloroform (Sigma Aldrich), methanol (Sigma Aldrich) and diH_2_O (Sigma Aldrich).

### 2.2 Materials, microbial strains and cell line

The strains used in this study were the Gram-negative *Pseudomonas aeruginosa* ATCC 27853, *Acinetobacter baumannii* ATCC 19606, *Escherichia coli* ATCC 25922; the Gram-positive *Staphylococcus aureus* ATCC 25923, *Staphylococcus epidermidis* ATCC 12228, *Bacillus megaterium* Bm11; the yeast *Candida albicans* ATCC 24433. The clinical isolates of *P. aeruginosa* were the strains R1 and 1Rm (Casciaro et al., 2017); the strains #2 and #3 (Luca et al., 2013); and the strain 19595 (Terri et al., 2022). Human immortalized keratinocytes (HaCaT cell line) were purchased from AddexBio (San Diego, CA, United States) and cultured in Dulbecco’s modified Eagle’s medium supplemented with 4 mM glutamine (DMEMg), 10% heat-inactivated fetal bovine serum (FBS), and 0.1 mg/mL of penicillin and streptomycin at 37°C and 5% CO_2_, in 25 cm^2^ or 75 cm^2^ flasks. All the other reagents used were purchased from Sigma-Aldrich (St. Luis, MO, United States).

### 2.3 Antimicrobial activity

The antimicrobial activity of KDEON WK-11 was evaluated by the microdilution broth method for the minimum inhibitory concentration (MIC) identification, as previously reported (Luca et al., 2013; Buommino et al., 2019). Aliquots of 50 μL of bacterial suspension in Mueller–Hinton broth (MH) in mid-log phase (concentration of 2 × 10^6^ cells/mL) were added to 50 μL MH containing serial two-fold dilutions of the peptide (from 0.375 to 100 μM), in a 96-well plate. For the yeast, Winge-broth and 5 × 10^4^ cells/mL were used (Buommino et al., 2019). The plate was then incubated for 16–18 h at 37°C (or 30°C for *Candida*) and the MIC was defined as the minimal concentration of peptide capable of visually inhibiting microbial growth (100% inhibition). MICs are reported as the modal value of at least three independent experiment. After incubation, for a representative Gram-positive, Gram-negative and yeast strain (i.e., *S. epidermidis* ATCC 12228, *P. aeruginosa* ATCC 27853 and *C. albicans* ATCC 24433), aliquots from the well corresponding to the MIC, 2 x MIC and 4 x MIC were spread on agar plates for colony forming unit (CFU) counting (Mangoni et al., 2008b). The bactericidal activity of KDEON WK-11 was finally assessed against *P. aeruginosa* ATCC 27853, in a condition that better mimics a physiological environment (i.e., the physiological phosphate buffered saline, PBS). The bacterial culture was grown until an optical density (O.D.) of 0.8 at λ = 590 nm was reached and then centrifuged (1,400 × g for 10 min) and resuspended in PBS. Aliquots of 100 μL (cell concentration of 1 × 10^6^ CFU/mL) were incubated with different concentrations of KDEON WK-11 (MIC, ½ x MIC and ¼ x MIC) for 5, 15, 30 and 60 min. At each time-point, aliquots were spread on agar plates for the CFU counting. Controls were cells treated with vehicle. Data are reported as the mean ± the standard error of the mean (SEM) of three independent experiments.

### 2.4 Sytox green assay

To assess the ability of KDEON WK-11 to perturb the cytoplasmic membrane of representative Gram-positive, Gram-negative and yeast strains (*S. epidermidis* ATCC 12228, *P. aeruginosa* ATCC 27853 and *C. albicans* ATCC 24433), the Sytox Green assay was performed as previously reported (Islas-Rodriguez et al., 2009). Approximately 1 × 10^7^ CFU/mL for bacteria and 1 × 10^6^ CFU/mL for yeast were incubated with 1 μM Sytox Green in PBS or 0.01 M sodium phosphate buffer (NaPB), respectively, for 5 min in the dark. After peptide addition, changes in fluorescence intensity (λ_exc_ = 485 nm, λ_ems_ = 535 nm) caused by the binding of the dye to intracellular DNA were monitored for 60 min in a microplate reader (Infinite M200, Tecan, Salzburg, Austria) at 37°C or 30°C for bacteria or yeast. Controls were cells treated with the vehicle PBS. The experiments were performed three times.

### 2.5 Antibiofilm activity

*P. aeruginosa* ATCC 27853 was grown at 37°C until an O.D. of 0.8 at λ = 590 nm was reached. Aliquots of 100 µL of bacteria (concentration of 1 × 10^6^ CFU/ml) in Luria Bertani (LB) were dispensed into the wells of a 96-multiwell plate, which was incubated for 20 h at 37°C to allow biofilm formation. Subsequently, the medium containing planktonic cells was aspirated from the wells and the wells were rinsed twice with 150 µL of PBS to remove any remaining non-adherent cells. After washing, each well was filled with PBS supplemented with different two-fold serial dilutions of KDEON WK-11. The plate was then incubated for 2 h at 37°C and, after treatment, the wells were rinsed twice with PBS. Finally, aliquots of 150 µL of 3-(4,5-dimethylthiazol-2-yl)-2,5-diphenyltetrazolium bromide (MTT) (0.5 mg/mL) were dispensed in each well to evaluate biofilm cell viability after 4 h incubation at 37°C. The reaction was stopped by adding sodium dodecyl sulfate (SDS) (final concentration of 5% v/v) and the absorbance of each well was recorded at 570 nm using the microplate reader. The percentage of biofilm viability was calculated with respect to the untreated samples, as previously reported (Luca et al., 2013). Data are reported as the mean ± standard error of the mean (SEM) of three independent experiments.

### 2.6 Inhibition of biofilm formation and pyoverdine production

The capability of KDEON WK-11 in inhibiting *P. aeruginosa* biofilm formation was assessed as previously reported, with some modifications (Casciaro et al., 2019; Corte et al., 2019). *P. aeruginosa* ATCC 27853 was grown at 37°C until an O.D. of 0.8 at λ = 590 nm was reached. Aliquots of 50 µL of bacteria (final cell concentration of 1 × 10^6^ CFU/mL) in Luria Bertani (LB) were dispensed into the wells of a 96-multiwell plate cointaining 50 µL of LB supplemented with different peptide concentrations. The plate was incubated for 20 h at 37°C to allow biofilm formation. After incubation, the absorbance of the microbial culture was read at λ = 590 nm with the microplate reader (Infinite M200, Tecan, Salzburg, Austria), to exclude that any inhibition of biofilm formation was simply due to a reduced bacterial growth at sub-MIC concentrations. The supernatants of the wells corresponding to 1/2, 1/4 and 1/8 x MIC were collected, centrifuged for 10 min at 12,000 x *g* to separate the bacterial pellet. The obtained supernatants were then analyzed for pyoverdine quantification by measuring the fluorescence intensity at 460 nm after excitation at 400 nm with the Tecan microplate reader, as previously reported (Deziel et al., 2001; Casciaro et al., 2019). Finally, the biomass quantification was measured as reported in (Casciaro et al., 2019): the wells were washed twice, then the biofilm was fixed with 99% methanol in water and stained with Crystal Violet (0.05% in water) for biomass evaluation. All the data obtained in these experiments are reported as mean ± SEM of three independent experiments.

### 2.7 Induction of resistance

The induction of resistance was conducted as previously reported (Casciaro et al., 2018). Briefly, multiple exposures of the bacterial suspension to serial two-fold dilutions of KDEON WK-11 were performed using a diluted inoculum (1:10,000 in MH) of the bacterial culture grown at ½ × MIC. After 15 cycles, bacteria were grown in drug-free LB. Afterwards, the standard MIC assay was carried out as described above. The final MICs were compared to the initial ones and graphed as fold increase in MIC. The polycationic antibiotic colistin was also tested for comparison. The experiment was conducted in two independent replicates.

### 2.8 Electrospray ionisation mass spectrometry

Electrospray Ionisation (ESI) mass spectrometry was performed at room temperature. A stock solution of 45%, chloroform 55% methanol and 0.5% diH_2_O was made and aliquots of 1 mL were taken in triplicate, for sample stock preparation. Sample stocks containing KDEON WK-11 and Lipid A were made and mixed in a 1:1 ratio, containing 0.005 mg/mL of peptide and Lipid A. Samples were run on Micro_TOF ESI-MS (Bruker Daltonics), FT-ICR-MS and Nanodrop-2000 UV-VIS Spectrometer.

### 2.9 Cytotoxicity *in vitro*

Cell viability was quantified by the colorimetric MTT assay performed as previously reported (Bellavita et al., 2021). Approximately 4 × 10^4^ cells resuspended in 100 μL of DMEMg plus 2% FBS were plated in each well of 96-well plates then incubated at 37°C and 5% CO_2_ for approximately 24 h. The medium was removed, a wash with 100 μL of serum-free DMEMg was performed and subsequently, 100 μL of fresh serum-free DMEMg containing the peptide at different concentrations was added in each well. As controls, HaCaT cells were treated with vehicle. After 24 h incubation at 37°C and 5% CO_2_, the culture medium was removed, each well was washed with 100 μL of Hank’s buffer (136 mM NaCl, 4.2 mM Na_2_HPO_4_, 4.4 mM KH_2_PO_4,_ 5.4 mM KCl, 4.1 mM NaHCO_3_, pH 7.2, supplemented wih 20 mM D-glucose) which was then replaced by 100 μL of 0.5 mg/mL MTT prepared in Hank’s buffer (Cappiello et al., 2016). The plate was incubated for 4 h under these same conditions. Finally, 100 μL of acidified isopropanol was added to each well, and absorbance was measured at 570 nm using the microplate reader. HaCaT cell viability was calculated with respect to the control and expressed as a percentage. Data are reported as the mean ± SEM of three independent experiments.

## 3 Results

### 3.1 The antimicrobial activity of KDEON

To assess the antimicrobial activity of KDEON WK-11 peptide (Table 1), its MIC values after 18 h of treatment were determined against a panel of Gram-positive and Gram-negative bacteria, and yeasts. As reported in Table 2, KDEON WK-11 showed notable antimicrobial activity against all the tested strains with MICs ranging from 0.75 to 50 μM. Regarding the Gram-positive bacteria, KDEON peptide had the strongest activity against *B. megaterium* Bm 11 (MIC of 0.75 μM) and *S. epidermidis* ATCC 12228 (MIC of 3.12 μM) while a weaker activity was displayed against the human pathogen *S. aureus* (MIC of 25 μM). When KDEON WK-11 was tested against Gram-negative bacteria, the weakest activity was detected against *A. baumannii* ATCC 19606 (MIC of 50 μM), while it showed a potent antimicrobial effect against *E. coli* ATCC 25922 and the human pathogen *P. aeruginosa* (MIC of 3.12 μM). A notable antimicrobial efficacy was also detected against *C. albicans* with a MIC of 6.25 μM. Considering the potent activity against *P. aeruginosa*, the activity of KDEON WK-11 was also tested against a panel of clinical isolates (Table 2): the antimicrobial activity of KDEON WK-11 was comparable to that obtained against the reference strain ATCC 27853 (i.e., MICs ranging from 1.56 to 6.25 μM).

**TABLE 1:** Primary structure, net charge at neutral pH, grand average of hydropathicity (GRAVY) and theoretical isoelectric point (pI) of KDEON WK-11.

Peptide	Sequence	Net charge	GRAVY	Theoretical pI
KDEON WK-11	WWKKWWKKWWK	+5	−2.264	10.60

GRAVY and theoretical pI values were provided by https://web.expasy.org.

**TABLE 2:** Antimicrobial activity of KDEON peptide, expressed as MIC values.

Strain	MIC (μM)
Gram-positives	
*Staphylococcus aureus* ATCC 25923	25
*Staphylococcus epidermidis* ATCC 12228	3.12
*Bacillus megaterium* Bm 11	0.75
Gram-negatives	
*Acinetobacter baumannii* ATCC 19606	50
*Escherichia coli* ATCC 25922	6.25
*Pseudomonas aeruginosa* ATCC 27853	3.12
*Pseudomonas aeruginosa* R1	1.56
*Pseudomonas aeruginosa* 1Rm	3.12
*Pseudomonas aeruginosa* #2	6.25
*Pseudomonas aeruginosa* #3	6.25
*Pseudomonas aeruginosa 19595*	3.12
Yeasts	
*Candida albicans* ATCC 24433	6.25

**FIGURE 1:**
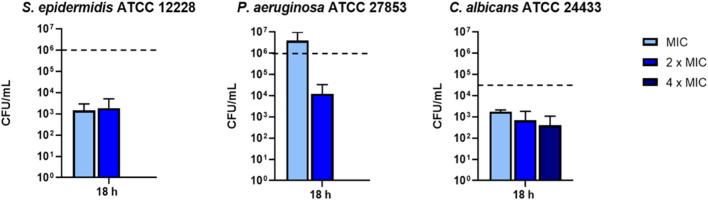
Bacterial cells viability expressed as CFU/mL after 18 h of peptide treatment. Dotted line represents initial inoculum density. Data represent the mean ± SEM of three independent experiments.

To verify whether the inhibitory effect of KDEON peptide was related to microbial killing, after MIC determination, aliquots from the well corresponding to MIC, 2 × MIC and 4 × MIC of three representative strains were spread onto LB agar plates for CFU counting. As reported in Figure 1, the strongest reduction of cell viability (>99%) was detected against *S. epidermidis* at the MIC and 2 × MIC, while a total killing (100%) occurred at 4 × MIC. Analogously, against *P. aeruginosa*, there was a reduction of approximately 99% of the initial inoculum at 2 × MIC, while total killing was recorded at 4 × MIC. In comparison, against *C. albicans,* KDEON peptide reduced >90% viability of the initial bacterial inoculum (dotted line) at MIC, 2xMIC and 4 × MIC.

### 3.2 Permeabilization of cell membrane

**FIGURE 2:**
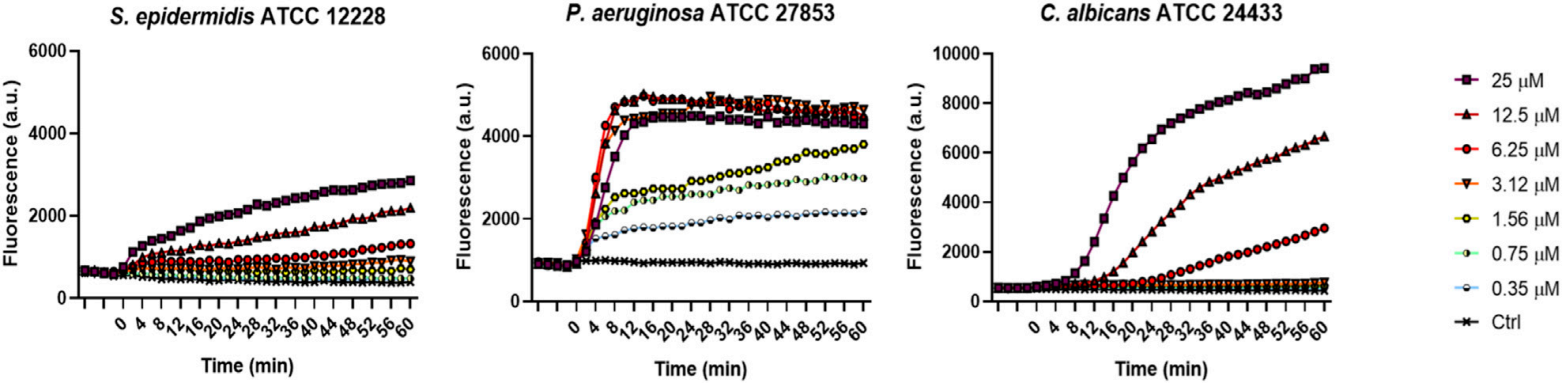
Membrane perturbation induced by KDEON as detected by the Sytox Green assay. Time 0 represents peptide addition, after stabilization of the fluorescence signal of the bacterial suspension in 1 μM Sytox Green. Values correspond to one representative experiment of three.

To investigate the membrane-perturbing activity of KDEON WK-11 as a plausible mechanism of antimicrobial action, we performed the Sytox Green assay. This assay assessed the capability of the peptide to destabilize the microbial cytoplasmic membrane, thus leading to the intracellular entry of the membrane impermeant fluorescent probe Sytox Green, with consequent increase of fluorescence intensity due to its binding to intracellular nucleic acids. As reported in Figure 2, KDEON WK-11 provoked a slow and weak perturbation of the membrane of the Gram-positive *S. epidermidis* strain, while a faster and stronger effect was recorded for *C. albicans* with an enhanced increase in fluorescence intensity, particularly at 25 and 12.5 μM after ∼10 min following peptide addition. Interestingly, a very fast kinetic was found for KDEON WK-11 at almost all concentrations used against *P. aeruginosa,* with the highest values of fluorescence achieved within the first minutes of peptide treatment.

**FIGURE 3:**
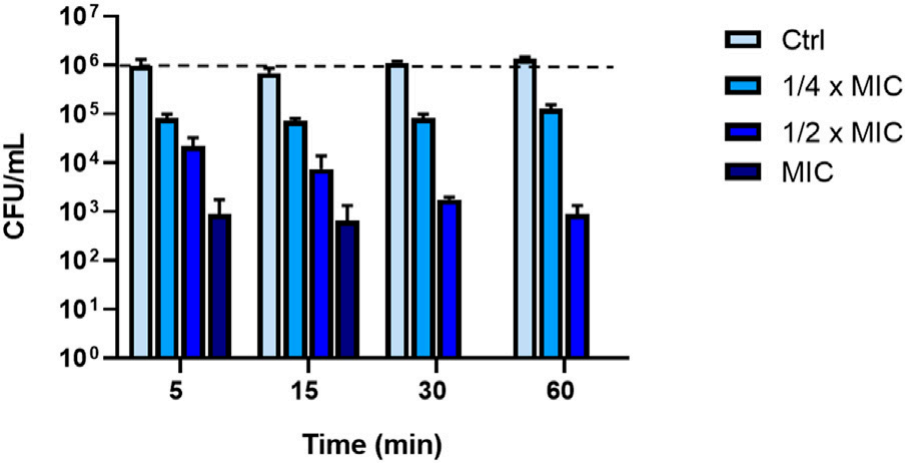
Killing kinetic of KDEON peptide against *P. aeruginosa* ATCC 27853 evaluated by CFU counting after 5, 15, 30 and 60 min of peptide treatment. Dotted line represents initial inoculum density. Data represent the mean ± SEM of three independent experiments.

To explore whether the fast kinetics of membrane permeabilization of *P. aeruginosa* was concomitant with cell death, a killing kinetics was conducted in PBS by testing three different peptide concentrations i.e., ¼ x MIC (0.75 μM); ½ x MIC (1.56 μM) and MIC (3.12 μM). This condition was essential to evaluate the effectiveness of the peptide in physiological solution, a remarkably aspect for its potential clinical application. As reported in Figure 3, KDEON WK-11 caused a reduction in CFU of more than 90% at ¼ x MIC after 5 min. At ½ x MIC, it provoked a reduction in CFU of more than 99% within 60 min, while at the MIC, KDEON WK-11 induced a notable and rapid reduction (approximately 99.9%) already after 5 min with a total killing at 30 min.

### 3.3 Antibiofilm activity of KDEON WK-11

**FIGURE 4:**
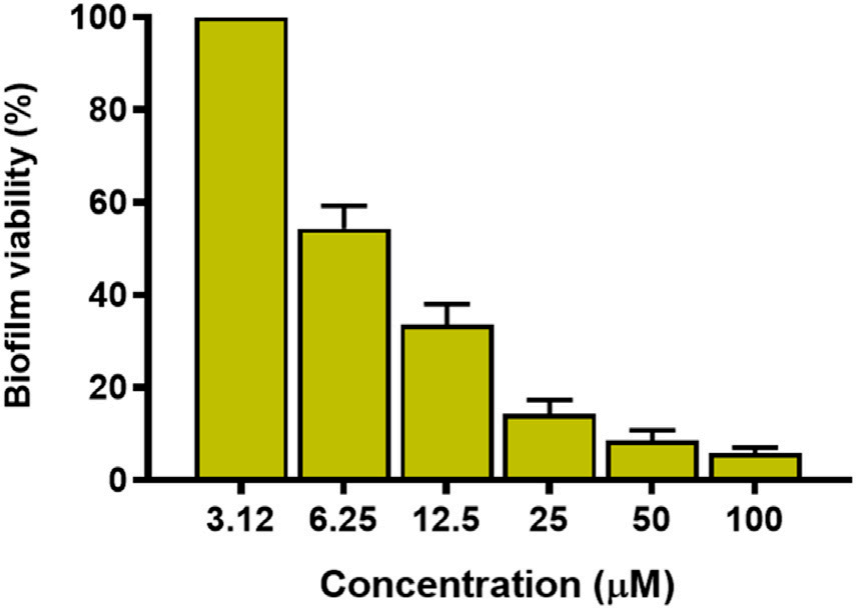
Metabolic activity of *P. aeruginosa* ATCC 27853 biofilm after treatment with KDEON WK-11 at concentrations of 3.12, 6.25, 12.5, 25, 50 and 100 μM for 2 h in PBS. Data represent the mean ± standard error of the mean (SEM) of three independent experiments.

Considering that *P. aeruginosa* can easily switch from a planktonic to sessile life-style and colonise biological and inert surfaces forming biofilms, the capability of KDEON WK-11 to disrupt and kill preformed biofilm was evaluated against the reference ATCC 27853 strain. Preformed biofilm was made as described in the Materials & Methods section and the peptide was tested at different concentrations for 2 h in PBS. As shown in Figure 4, KDEON WK-11 was active against *P. aeruginosa* biofilm and at 6.25 μM, reduced approximately 40% of biofilm metabolic activity. Of particular significance, when tested at 25, 50 and 100 μM the percentage of biofilm metabolic activityy was lower than 20%.

**FIGURE 5:**
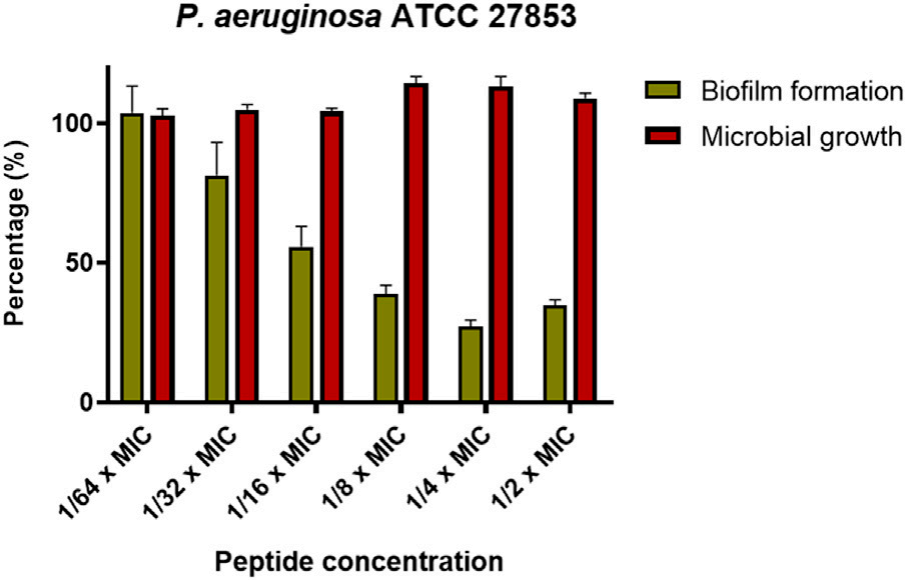
Percentage of biofilm biomass at different sub-MIC concentration of KDEON WK-11 (gold bars) in comparison with the microbial growth (red bars). Biofilm biomass was quantified by CV staining, while the microbial growth was evaluated by reading the absorbance at 590 nm. Data represent the mean ± SEM of three independent experiments.

Another important biological property of some antimicrobials is the ability to inhibit the formation of biofilms. KDEON WK-11 was tested for this capability against the reference strain *P. aeruginosa* ATCC 27853. As reported in Figure 5, when used at concentrations ranging from 1/2 to 1/32 of its MIC, KDEON WK-11 was capable of inhibiting the formation of biofilm after 20 h of treatment, with a reduction in biomass ranging from ∼60 to ∼40%. No antibiofilm activity was found at 1/64 of the MIC. As reported in Figure 5, at concentrations equal to 1/2, 1/4 and 1/8 of MIC, the biomass reduction is higher than 50%. Note also that such reduction is not linked to a reduction in the growth of the planktonic form of the bacterium, as demonstrated by the invariant absorbance values of the bacterial culture treated with the peptide at sub-MICs compared to the untreated control samples.

**FIGURE 6:**
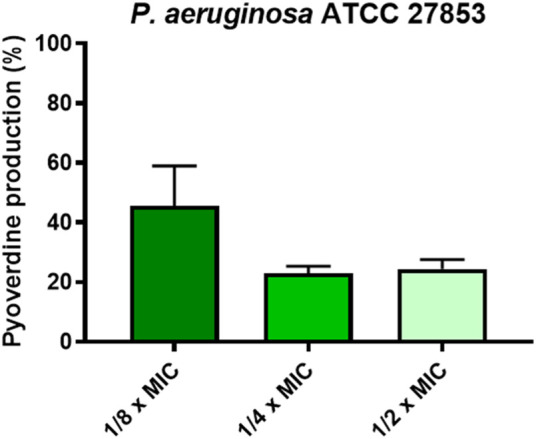
Percentage of pyoverdine production at 1/2, 1/4, and 1/8 x MIC of KDEON WK-11. Data represent the mean ± SEM of three independent experiments.

Finally, we also evaluated the effect of the peptide on the production of virulence factors i.e., pyoverdine, the primary siderophore of *P. aeruginosa*. As reported in Figure 6, at the concentrations at which the peptide is capable of inhibiting biofilm formation by more than 50% (i.e., 1/2, 1/4, 1/8 x MIC), KDEON WK-11 was also capable of reducing the production of pyoverdine by about 75% at 1/2 and 1/4 of the MIC and by about 50% at 1/8 of the MIC.

### 3.4 Induction of resistance by KDEON WK-11

**FIGURE 7:**
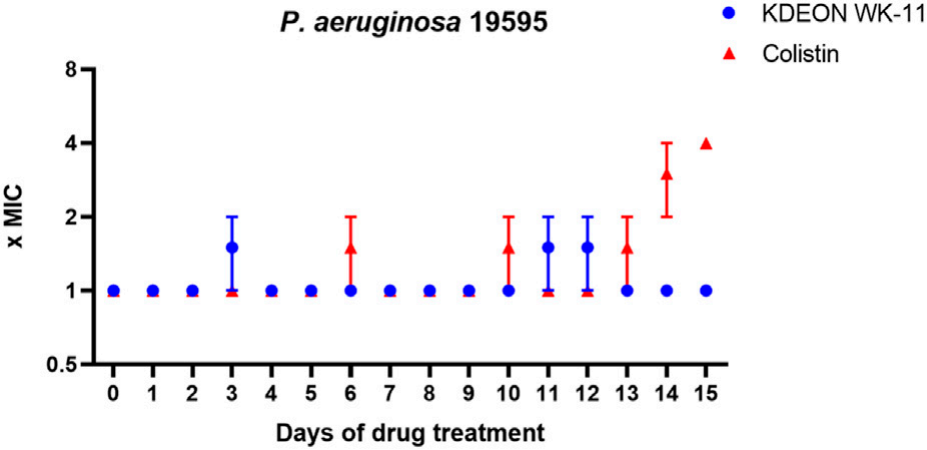
Fold increase in MIC of KDEON WK-11 and colistin against the clinical isolate *P. aeruginosa* 19595 after 15 cycles of treatment. The experiment was conducted in two independent replicates. The value represent the mean ± standard deviation (SD) of the fold increase in MIC of these two replicates. When SD is 0, the MIC values of both replicates were the same.

The induction of resistance was conducted on the clinical isolate *P. aeruginosa* 19595, using the antibiotic colistin as a reference. As reported in Figure 7, KDEON WK-11 did not provoke any increase in the MIC values after multiple exposures to the peptide, while colistin MIC became to 4-fold higher than the initial value after 15-days-treatment.

### 3.5 Electrospray ionisation mass spectroscopy

**FIGURE 8:**
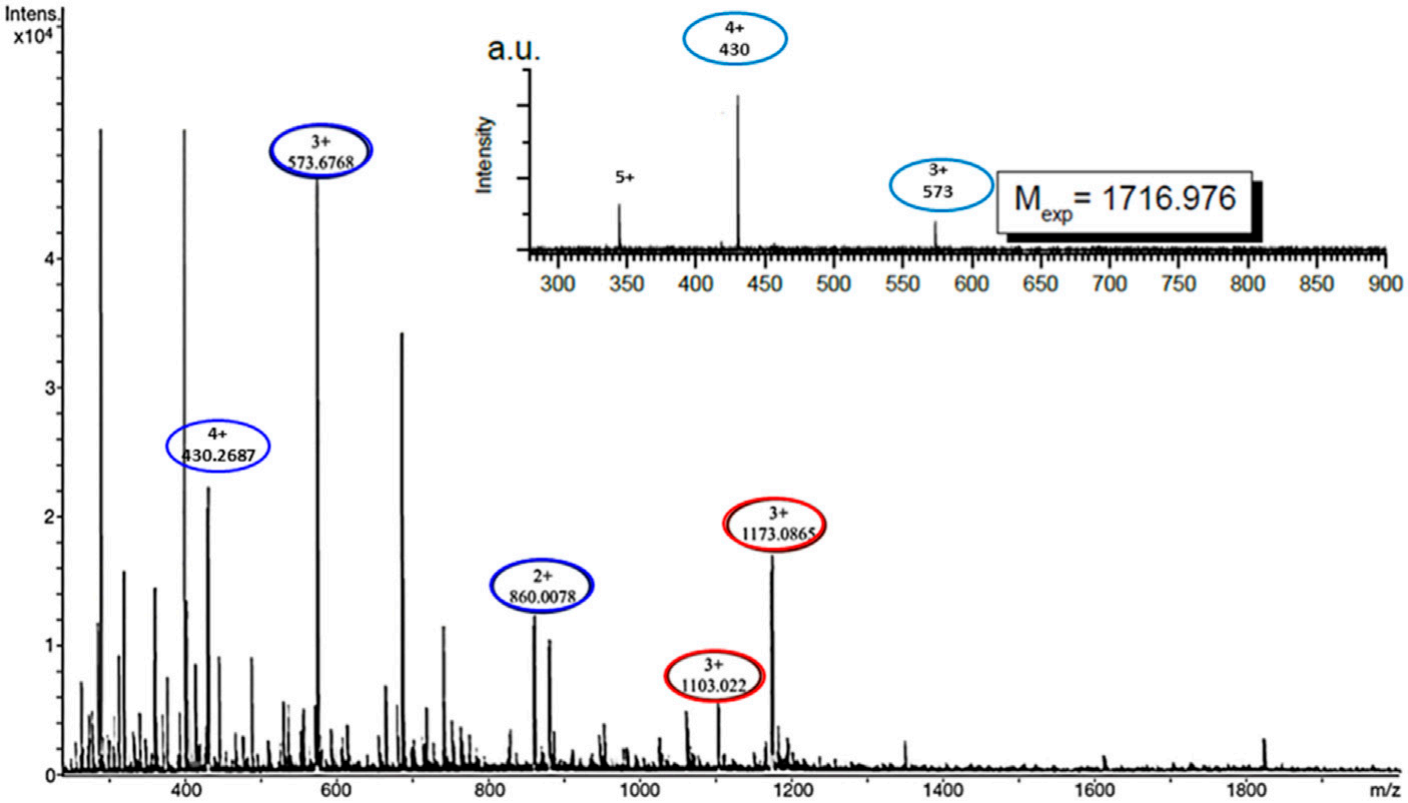
Electrospray ionisation mass spectra showing Lipid A binding to KDEON WK-11 represented by ion complex formation seen as single peaks expressing the molecular mass of both components. Red circles present at peaks 1103 and 1173 indicate the presence of the peptide-ion complex, proving the binding of KDEON WK-11. Blue circles at peaks 430, 573 and 860 (m/z), indicate the peptide alone (4+, 3+, 2 + charge state respectively of WK11), showing the significant difference between the two, in the presence and absence of ion complex formation. The inset indicates the spectrum of the peptide alone.

**FIGURE 9:**
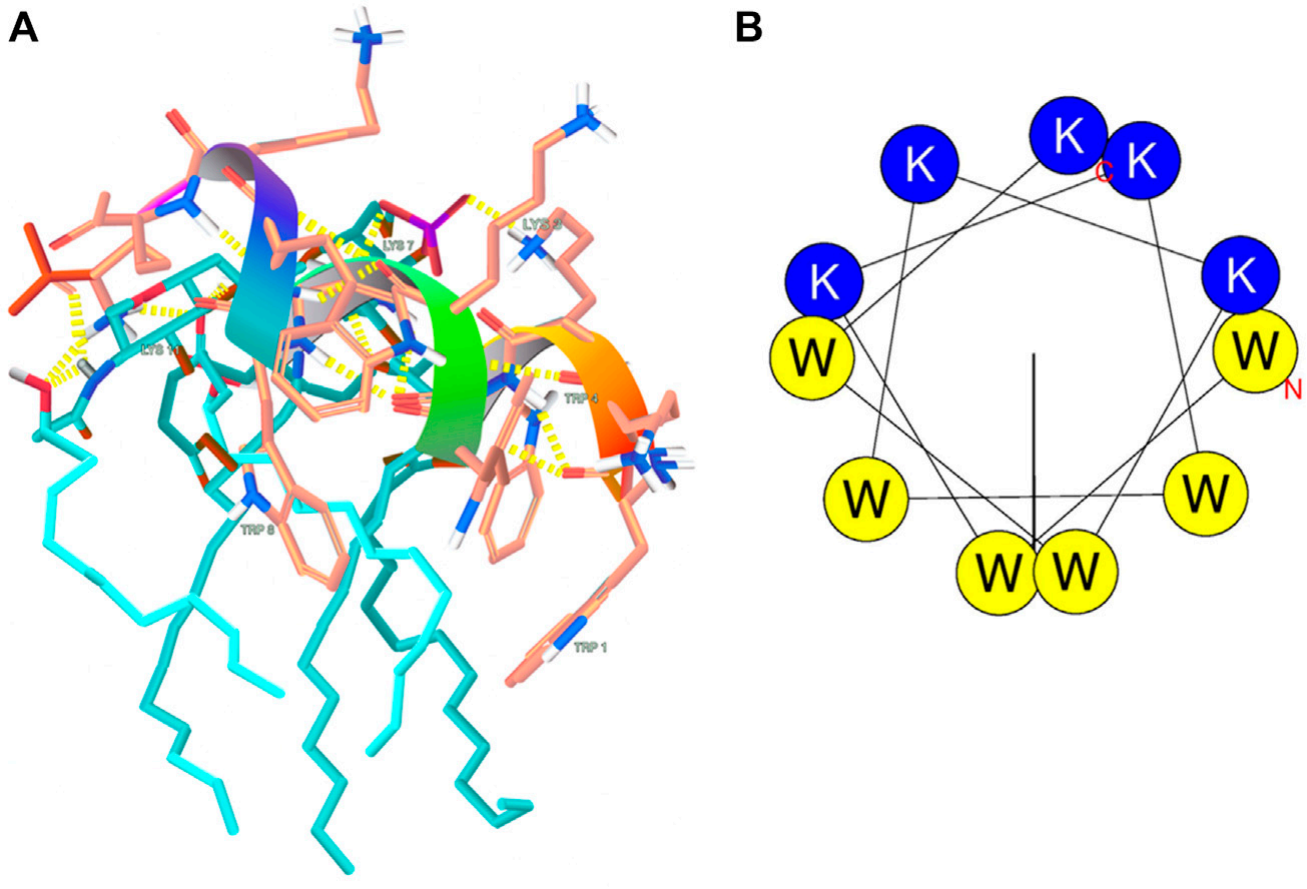
**(A)** Predicted LPS-binding of KDEON WK-11; **(B)** Structural predictions showing the amphipathic character of the helical wheel projection of KDEON WK-11. Blue and yellow colors indicate basic hydrophilic and hydrophobic residuces respectively.

ESI-MS experiments were carried out on lipid A and KDEON WK-11 under a variety of solution and mass spectrometry conditions. The results of MS methods showed binding of diphosphorylated-lipid A to KDEON WK-11, as significant ion (Lipid A-peptide) complexes were detected from CHL/MeOH/H_2_O solution (Figure 8). These peaks were visible at 1103 and 1173, when compared to peaks representing the peptide alone, at 573 and 860. This was also supported by independent structural predictions generated by Mobyle@RPBS (University of Paris) showing that KDEON WK-11 conforms in an alpha-helix as its secondary structure when complexed to Lipid A (Figure 9A). From these structural predictions, we could also visualize the amphipathic character of the alpha-helix structure of KDEON WK-11, vital for carrying out its antimicrobial activity (Figure 9B).

### 3.6 Cytotoxicity *in vitro*

Due to the ability of *P. aeruginosa* to easily infect skin wounds, we tested the cytotoxicity of the peptide by the MTT assay against a human cell line of keratinocytes, the principal type of cell found in the *epidermis*, the outermost layer of the skin.

**FIGURE 10:**
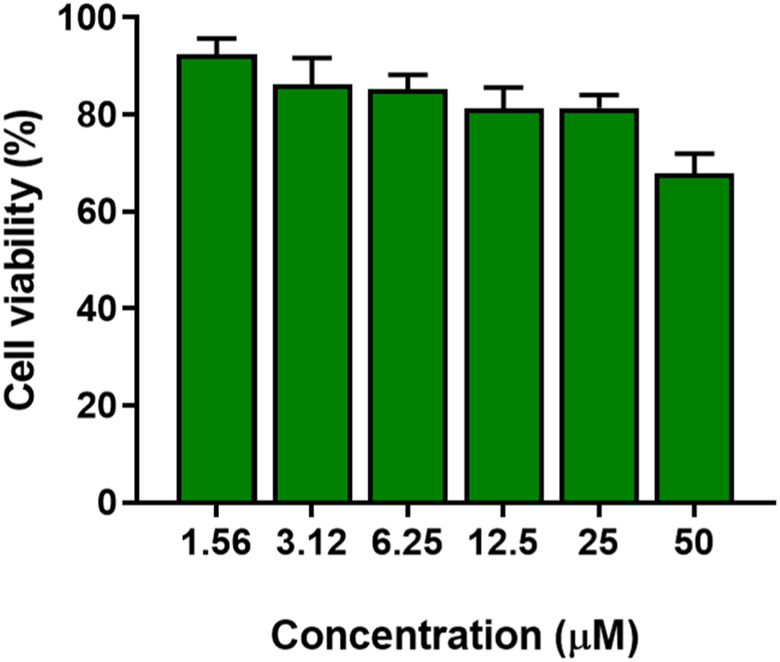
Cytotoxicity of KDEON WK-11 as expressed in percentage cell viability assessed by the MTT assay testing varying concentrations of peptide from 1.56 to 50 μM. Data represent the mean ± SEM of three independent experiments.

As reported in Figure 10, the peptide did not show significant toxicity at concentrations below 25 μM, with reduction in cell viability less than 20%. At a concentration of 50 μM (16-fold higher the MIC against *Pseudomonas* (3.12 μM), cell viability was reduced by approximately 25%.

## 4 Discussion

Naturally-occurring AMPs represent an interesting class of molecules with different biological functions and can serve as a template for the “*de novo*'' design and development of new peptides. One such bioactive peptide with multiple functions is lactoferrin, and several studies have aimed to design lactoferrin-derived peptides for modulating these activities (Gifford et al., 2005; Sinha et al., 2013; Kim et al., 2016; Gruden and Poklar Ulrih, 2021). Many peptides, particularly those derived from lactoferrin or similar origin, are composed of sequences that cover two characteristics. They include a hydrophobic, or lipophilic component, and a cationic charge (Chen et al., 2003). Previous studies on the sequences required for efficacious antimicrobial activity have established the importance of repetitive subunits of residues, and the most effective number of repeats (Deslouches et al., 2016). While many studies have described that increasing peptide chain length corresponded to an increase in antimicrobial activity, it appears that at a certain length, a threshold is reached, above which increasing the number of residues in the AMP chain can hinder activity (Chen et al., 2003; Ahn et al., 2017; Ramamourthy et al., 2020). Based on these findings, we established that 11-residues was likely to be an effective chain length for optimum antimicrobial activity.

Taking these considerations into account, we designed an 11-residue long peptide, named KDEON WK-11 that consists of the repetition of two residues (Lys and Trp), abundant in the primary structure of lactoferrin, and assessed its antimicrobial activity, with particular emphasis on its antipseudomonal properties. Lysine, as a cationic, basic residue plays a role in the electrostatic interaction between AMP and anionic cell membrane (Jin et al., 2016; Arias et al., 2018; Ramamourthy et al., 2020). Lys can be commonly found in naturally occurring AMPs and when contrasted with other basic residues used in alternative sequences, Lys produces less cytotoxic effects on host cells which may be due to its improved selectivity, preventing the haemolysis of healthy red blood cells (Gopal et al., 2013; Jin et al., 2016). There is some contest between Lys and Arg as to which is the more favourable cationic residue (Han et al., 2016; Krishnan et al., 2021). Some authors report that Arg containing peptides may induce a more potent antimicrobial effect as it can more easily perturb and incorporate into bacterial cell membranes (Andreev et al., 2014; Deslouches et al., 2016). These effects can be attributed to the guanidinium side chain on the structure of Arg, forming numerous hydrogen bonds with phosphate groups (Tang et al., 2008; Gopal et al., 2013). However, with increased antimicrobial activity, there is a corresponding increase in haemolytic and cytotoxic effects at therapeutic concentrations (Yang et al., 2003; Nan et al., 2010) and so, in many cases, Lys is the safer choice. In addition, some studies have speculated that the Lys residue may play a role in carrying out its antimicrobial effect by interfering with the phosphodiester bonds of bacterial DNA, or RNA, through its positively charged side chains. For this reason, and its improved safety profile, Lys was the more appropriate amino acid to include, in order to enhance the AMPs mechanism of antimicrobial activity (Travis et al., 2000; Nan et al., 2011; Luong et al., 2018).

Tryptophan is a hydrophobic residue involved in the permeabilization and disruption of the bacterial cell membrane (Nan et al., 2009; Rajasekaran et al., 2015; Lyu et al., 2019). Hydrophobic residues can insert into the hydrophobic core of the lipid bilayer, influencing pore size and stability of the membrane (Bi et al., 2013; Bi et al., 2014). The structure of tryptophan contains an aromatic indole side chain that can improve peptide anchoring and penetration *via* hydrogen bonding with phospholipid head groups. (Gopal et al., 2013; Ramamourthy et al., 2020). AMPs containing Trp residues near to the N-terminal have a higher propensity to alpha-helix formation, which can additionally improve interactions with the hydrophobic core of the lipid bilayer, (Bi et al., 2013; Zhu et al., 2014; Shagaghi et al., 2016), and was proven by predicted structural analysis (Figure 9). The role of the Trp residue may explain the increased potency of bovine lactoferrin, containing two Trp residues, when compared to human lactoferrin, containing only one (Vogel et al., 2002).

Previous work has established Trp as an essential residue in AMP sequences that target *P. aeruginosa*, capable of perturbing the inner bacterial membrane and expressing anti-biofilm properties (Zhu et al., 2014). Based on these findings, we assessed the antimicrobial activity of KDEON WK-11 against a variety of Gram-positive and Gram-negative bacterial strains. KDEON WK-11 has a broad spectrum of activity with MIC values ranging from 0.75 to 50 μM. As expected, the peptide showed significant activity against the human pathogen *P. aeruginosa* ATCC 27853 (MIC of 3.12 μM, Table 2) and a panel of clinical isolates (MICs ranging from 1.56 to 6.25 μM, Table 2). Interestingly, the peptide showed the ability to decrease the metabolic activity of *Pseudomonas* preformed biofilm (at concentrations ranging from 6.25 to 100 μM) and to inhibit its formation even at concentrations significantly lower than the MIC (Figures 4, 5), influencing also the production of the virulence factor pyoverdine (Figure 6). As for many other AMPs, its antimicrobial activity was expressed through the destabilization of the plasma membrane of the bacterium, proved by the Sytox Green assay (Figure 2). In agreement with the fast membrane perturbation kinetics was the killing process with a two log reduction in the number of viable bacteria within the first 5 min of peptide treatment at the MIC, suggesting membrane perturbation as the plausible mechanism of bactericidal activity.

When comparing KDEON WK-11 to previously established AMPs with potent activity against *P. aeruginosa,* we found that MIC values were certainly comparable and, in some cases, superior to alternative sequences. For example, one sequence created by Bi et al. was developed to emphasise the importance of the Trp residue in the peptide sequence, and contained both Trp and Lys in an AMP 13 residues long. It expressed an MIC against *P. aeruginosa* of 4.69 μM (Bi et al., 2013). The study concluded that in addition to the number of Trp residues, the position of such residues were vital for activity and that by including Trp residues in pairs, similar to KDEON WK-11, the hydrophobic face of the sequence could interact more significantly with the interfacial region of the lipid membrane. The AMP P5 similarly features Lys in a sequence 13 residues long andexpressed activity against *P. aeruginosa* with an MIC of 6.25 μM (Kwon et al., 2019). The peptide produced a positive safety profile, with no significant cytotoxicity due to the use of Lys residues and additionally, replicated the KDEON WK-11 peptide in its alpha-helix formation and in capabilities of binding to LPS or lipoteichoic acid, on the bacterial membrane of Gram-negative or Gram-positive bacteria, respectively. Interestingly, another comparable sequence was developed by repeating the Trp and Lys residues intermittently, showing similarities to KDEON WK-11 with multiple analogues created for various numbers of repeats (Ramamourthy et al., 2020). For chain lengths of 8–10 residues long, these sequences produced MICs between 3.12 and 25 μM. Similarly, one sequence developed by Rosenfeld et al. intermittently repeated Lys and Leu residues in various AMPs approximately 12 residues long (Rosenfeld et al., 2010). The paper demonstrated that the antibacterial activity of the peptides, correlated directly with hydrophobicity and that the optimum number of Lys residues contained in a 12 residue structure, was 5, replicating the presence of Lys residues in the sequence of KDEON WK-11. By repeating the two residues, the peptide balances hydrophobic and hydrophilic characteristics and can optimally interact with the bacterial membrane to exert its antimicrobial activity. By considering the mechanisms and underlying principles behind alternative peptide sequences active against *P. aeruginosa,* KDEON WK-11 was successfully designed to optimise characteristics of these peptides and to produce significant antimicrobial effects against the Gram-negative bacteria.

Considering that bacterial infections such as those induced by *Pseudomonas* have started to develop resistance against traditional antimicrobials, the broader applications of a successful agent should be considered. Interestingly, when tested against the clinical isolate *P. aeruginosa* 19595, KDEON WK-11 did not elicit resistance against it, when compared against the conventional antibiotic colistin, after 15 cycle of drug exposure (Figure 7). Sepsis and septic shock can occur when bacterial cells are rapidly dividing and during periods of exponential growth, as well as after death, cells release endotoxin or LPS, which can bind to the endothelial lining of the circulatory system (Mangoni et al., 2008a; Gotts and Matthay, 2016). This interaction can trigger an inflammatory reaction, resulting in sepsis, which without sufficient treatment can develop into septic shock and in many cases, can be fatal (Giacometti et al., 2006).

Taking these factors into account, we considered whether KDEON WK-11 may be an appropriate agent to counter the immune response that occurs in sepsis, by binding to the portion of LPS known as lipid A (Schmitt et al., 2016). If KDEON WK-11 reduces the freely circulating LPS, prevening its binding to the endothelium, it would be possible to reduce the immune response that drives sepsis and septic shock (Giacometti et al., 2006; Mangoni and Shai, 2009; Gustafsson et al., 2010; Wei et al., 2013; Rajasekaran et al., 2015; Kumari et al., 2020; Nagaoka et al., 2020). Japelj et al. previously designed an 11-residue long lactoferrin-derived peptide with endotoxin binding abilities (Japelj et al., 2005). Conforming into a “T” shape, the peptide consisted of a hydrophobic core and contained two clusters of basic residues that interact with the two phosphate groups of lipid A. In addition, previous studies have established a link between chain length and potential for LPS binding, specifying the repetition of amino acids (peptide length of 10 residues) which produced the most favorable LPS-binding activity, when compared to various alternative chain lengths (Travis et al., 2000; Gopal et al., 2013). Using Electrospray Ionisation mass spectrometry, we proved the binding of KDEON WK-11 to lipid A. Indeed, visible peaks corresponding to the molecular mass of lipid A, KDEON WK-11 and their complexes were visible on the mass spectra. The binding was corroborated by predictive studies showing a secondary alpha helix structure of the peptide when bound to LPS (Figure 9A) and that is expected to facilitate the peptide insertion into the lipid bilayer of membranes (Khara et al., 2017; Smirnova et al., 2020). This is also in line with previous studies on the LPS-neutralization effect by the α-helical AMP Esc(1-21) showing its interaction with the negatively charged phosphate groups of lipid A (Mangoni et al., 2008a; Ghosh et al., 2016). Note also that the α-helix conformation is one of the key features of the ability of AMP to permeabilize biological membranes and to exert bactericidal but also cytotoxic activity (Oren et al., 2002; Zelezetsky and Tossi, 2006). Nevertheless, KDEON WK-11 showed a safe profile up to 50 μM (cell viability >75%). Overall these results make the KDEON peptide an attractive lead compound to exhibit both anti-LPS/anti-sepsis and antibacterial activity.

## 5 Conclusion

The peptide sequence developed as KDEON WK-11 clearly expresses antimicrobial and anti-biofilm activity, particularly against *Pseudomonas* without provoking cytotoxicity at antibacterial concentrations. Furthermore, the promising predictive conformational studies have encouraged continued efforts to determine its anti-sepsis properties and the future role of KDEON WK-11 as either an antimicrobial or anti-sepsis agent.

## Data Availability

The original contributions presented in the study are included in the article/Supplementary Material, further inquiries can be directed to the corresponding author.

## References

[B1] Ahn M. Gunasekaran P. Rajasekaran G. Kim E. Y. Lee S. J. Bang G. (2017). Pyrazole derived ultra-short antimicrobial peptidomimetics with potent anti-biofilm activity. Eur. J. Med. Chem. 125, 551–564. 10.1016/j.ejmech.2016.09.071 27718471

[B2] Andreev K. Bianchi C. Laursen J. S. Citterio L. Hein-Kristensen L. Gram L. (2014). Guanidino groups greatly enhance the action of antimicrobial peptidomimetics against bacterial cytoplasmic membranes. Biochim. Biophys. Acta - Biomembr. 1838, 2492–2502. 10.1016/j.bbamem.2014.05.022 PMC4125507 24878450

[B3] Appelmelk B. J. An Y. Q. Geerts M. Thijs B. G. De Boer H. A. Maclaren D. M. (1994). Lactoferrin is a lipid A-binding protein. Infect. Immun. 62, 2628–2632. 10.1128/iai.62.6.2628-2632.1994 8188389 PMC186557

[B4] Arias M. Piga K. B. Hyndman M. E. Vogel H. J. (2018). Improving the activity of trp-rich antimicrobial peptides by arg/lys substitutions and changing the length of cationic residues. Biomolecules 8, 19. 10.3390/biom8020019 29671805 PMC6023086

[B5] Bellavita R. Casciaro B. Di Maro S. Brancaccio D. Carotenuto A. Falanga A. (2021). First-in-Class cyclic temporin L analogue: Design, synthesis, and antimicrobial assessment. J. Med. Chem. 64, 11675–11694. 10.1021/acs.jmedchem.1c01033 34296619 PMC8389922

[B6] Bi X. Wang C. Ma L. Sun Y. Shang D. (2013). Investigation of the role of tryptophan residues in cationic antimicrobial peptides to determine the mechanism of antimicrobial action. J. Appl. Microbiol. 115, 663–672. 10.1111/jam.12262 23710779

[B7] Bi X. Wang C. Dong W. Zhu W. Shang D. (2014). Antimicrobial properties and interaction of two Trp-substituted cationic antimicrobial peptides with a lipid bilayer. J. Antibiot. 67, 361–368. 10.1038/ja.2014.4 24496141

[B8] Buommino E. Carotenuto A. Antignano I. Bellavita R. Casciaro B. Loffredo M. R. (2019). The outcomes of decorated prolines in the discovery of antimicrobial peptides from temporin-L. ChemMedChem 14, 1283–1290. 10.1002/cmdc.201900221 31087626

[B9] Cappiello F. Di Grazia A. Segev-Zarko L. A. Scali S. Ferrera L. Galietta L. (2016). Esculentin-1a-Derived peptides promote clearance of *Pseudomonas aeruginosa* internalized in bronchial cells of cystic fibrosis patients and lung cell migration: Biochemical properties and a plausible mode of action. Antimicrob. Agents Chemother. 60, 7252–7262. 10.1128/aac.00904-16 27671059 PMC5119005

[B10] Casciaro B. Dutta D. Loffredo M. R. Marcheggiani S. Mcdermott A. M. Willcox M. D. (2017). Esculentin-1a derived peptides kill *Pseudomonas aeruginosa* biofilm on soft contact lenses and retain antibacterial activity upon immobilization to the lens surface. Peptide Science 110, e23074. 10.1002/bip.23074 29086910

[B11] Casciaro B. Loffredo M. R. Luca V. Verrusio W. Cacciafesta M. Mangoni M. L. (2018). Esculentin-1a derived antipseudomonal peptides: Limited induction of resistance and synergy with aztreonam. Protein Pept. Lett. 25, 1155–1162. 10.2174/0929866525666181101104649 30381056

[B12] Casciaro B. Lin Q. Afonin S. Loffredo M. R. De Turris V. Middel V. (2019). Inhibition of *Pseudomonas aeruginosa* biofilm formation and expression of virulence genes by selective epimerization in the peptide Esculentin-1a(1-21)NH2. FEBS J. 286, 3874–3891. 10.1111/febs.14940 31144441 PMC6779485

[B13] Casciaro B. Cappiello F. Loffredo M. R. Ghirga F. Mangoni M. L. (2020). The potential of frog skin peptides for anti-infective therapies: The case of esculentin-1a(1-21)NH2. Curr. Med. Chem. 27, 1405–1419. 10.2174/0929867326666190722095408 31333082

[B14] Chen P. W. Shyu C. L. Mao F. C. (2003). Antibacterial activity of short hydrophobic and basic-rich peptides. Am. J. Vet. Res. 64, 1088–1092. 10.2460/ajvr.2003.64.1088 13677384

[B15] Corte L. Casagrande Pierantoni D. Tascini C. Roscini L. Cardinali G. (2019). Biofilm specific activity: A measure to quantify microbial biofilm. Microorganisms 7, 73. 10.3390/microorganisms7030073 30866438 PMC6463164

[B16] Deslouches B. Hasek M. L. Craigo J. K. Steckbeck J. D. Montelaro R. C. (2016). Comparative functional properties of engineered cationic antimicrobial peptides consisting exclusively of tryptophan and either lysine or arginine. J. Med. Microbiol. 65, 554–565. 10.1099/jmm.0.000258 27046192 PMC5042116

[B17] Deziel E. Comeau Y. Villemur R. (2001). Initiation of biofilm formation by *Pseudomonas aeruginosa* 57RP correlates with emergence of hyperpiliated and highly adherent phenotypic variants deficient in swimming, swarming, and twitching motilities. J. Bacteriol. 183, 1195–1204. 10.1128/JB.183.4.1195-1204.2001 11157931 PMC94992

[B18] Doi Y. Bonomo R. A. Hooper D. C. Kaye K. S. Johnson J. R. Clancy C. J. (2017). Gram-negative bacterial infections: Research priorities, accomplishments, and future directions of the antibacterial resistance leadership group. Clin. Infect. Dis. 64, S30–s35. 10.1093/cid/ciw829 28350901 PMC5848311

[B19] Farnaud S. Evans R. W. (2003). Lactoferrin--a multifunctional protein with antimicrobial properties. Mol. Immunol. 40, 395–405. 10.1016/s0161-5890(03)00152-4 14568385

[B20] Ghosh A. Bera S. Shai Y. Mangoni M. L. Bhunia A. (2016). NMR structure and binding of esculentin-1a (1-21)NH2 and its diastereomer to lipopolysaccharide: Correlation with biological functions. Biochim. Biophys. Acta - Biomembr. 1858, 800–812. 10.1016/j.bbamem.2015.12.027 26724203

[B21] Giacometti A. Cirioni O. Ghiselli R. Mocchegiani F. Orlando F. Silvestri C. (2006). Interaction of antimicrobial peptide temporin L with lipopolysaccharide *in vitro* and in experimental rat models of septic shock caused by gram-negative bacteria. Antimicrob. Agents Chemother. 50, 2478–2486. 10.1128/aac.01553-05 16801429 PMC1489763

[B22] Gifford J. L. Hunter H. N. Vogel H. J. (2005). Lactoferricin: a lactoferrin-derived peptide with antimicrobial, antiviral, antitumor and immunological properties. Cell. Mol. Life Sci. 62, 2588–2598. 10.1007/s00018-005-5373-z 16261252 PMC11139180

[B23] Gopal R. Seo C. H. Song P. I. Park Y. (2013). Effect of repetitive lysine-tryptophan motifs on the bactericidal activity of antimicrobial peptides. Amino Acids 44, 645–660. 10.1007/s00726-012-1388-6 22914980 PMC3549253

[B24] Gotts J. E. Matthay M. A. (2016). Sepsis: pathophysiology and clinical management. Bmj 353, i1585. 10.1136/bmj.i1585 27217054

[B25] Gruden Š. Poklar Ulrih N. (2021). Diverse mechanisms of antimicrobial activities of lactoferrins, lactoferricins, and other lactoferrin-derived peptides. Int. J. Mol. Sci. 22, 11264. 10.3390/ijms222011264 34681923 PMC8541349

[B26] Gustafsson A. Olin A. I. Ljunggren L. (2010). LPS interactions with immobilized and soluble antimicrobial peptides. Scand. J. Clin. Lab. Invest. 70, 194–200. 10.3109/00365511003663622 20233038

[B27] Han H. M. Gopal R. Park Y. (2016). Design and membrane-disruption mechanism of charge-enriched AMPs exhibiting cell selectivity, high-salt resistance, and anti-biofilm properties. Amino Acids 48, 505–522. 10.1007/s00726-015-2104-0 26450121

[B28] Hellman J. Warren H. S. (1999). Antiendotoxin strategies. Infect. Dis. Clin. North Am. 13, 371–386. 10.1016/s0891-5520(05)70080-5 10340172

[B29] Islas-Rodriguez A. E. Marcellini L. Orioni B. Barra D. Stella L. Mangoni M. L. (2009). Esculentin 1-21: a linear antimicrobial peptide from frog skin with inhibitory effect on bovine mastitis-causing bacteria. J. Pept. Sci. 15, 607–614. 10.1002/psc.1148 19507197

[B30] Japelj B. Pristovsek P. Majerle A. Jerala R. (2005). Structural origin of endotoxin neutralization and antimicrobial activity of a lactoferrin-based peptide. J. Biol. Chem. 280, 16955–16961. 10.1074/jbc.m500266200 15687491

[B31] Jin L. Bai X. Luan N. Yao H. Zhang Z. Liu W. (2016). A designed tryptophan- and lysine/arginine-rich antimicrobial peptide with therapeutic potential for clinical antibiotic-resistant Candida albicans vaginitis. J. Med. Chem. 59, 1791–1799. 10.1021/acs.jmedchem.5b01264 26881456

[B32] Kaye K. S. Pogue J. M. (2015). Infections caused by resistant gram-negative bacteria: Epidemiology and management. Pharmacotherapy. 35, 949–962. 10.1002/phar.1636 26497481

[B33] Khara J. S. Obuobi S. Wang Y. Hamilton M. S. Robertson B. D. Newton S. M. (2017). Disruption of drug-resistant biofilms using de novo designed short α-helical antimicrobial peptides with idealized facial amphiphilicity. Acta Biomater. 57, 103–114. 10.1016/j.actbio.2017.04.032 28457962

[B34] Kim W. S. Kim P. H. Shimazaki K. (2016). Sensitivity of *Pseudomonas syringae* to bovine lactoferrin hydrolysates and identification of a novel inhibitory peptide. Korean J. Food Sci. Anim. Resour. 36, 487–493. 10.5851/kosfa.2016.36.4.487 27621689 PMC5018508

[B35] Krishnan M. Choi J. Choi S. Kim Y. (2021). Anti-endotoxin 9-meric peptide with therapeutic potential for the treatment of endotoxemia. J. Microbiol. Biotechnol. 31, 25–32. 10.4014/jmb.2011.11011 33263333 PMC9705858

[B36] Kumari T. Verma D. P. Afshan T. Verma N. K. Pant G. Ali M. (2020). A noncytotoxic temporin L analogue with *in vivo* antibacterial and antiendotoxin activities and a nonmembrane-lytic mode of action. ACS Infect. Dis. 6, 2369–2385. 10.1021/acsinfecdis.0c00022 32786286

[B37] Kwon J. Y. Kim M. K. Mereuta L. Seo C. H. Luchian T. Park Y. (2019). Mechanism of action of antimicrobial peptide P5 truncations against *Pseudomonas aeruginosa* and *Staphylococcus aureus* . Amb. Express 9, 122. 10.1186/s13568-019-0843-0 31363941 PMC6667604

[B38] Lizzi A. R. Carnicelli V. Clarkson M. M. Di Giulio A. Oratore A. (2009). Lactoferrin derived peptides: mechanisms of action and their perspectives as antimicrobial and antitumoral agents. Mini Rev. Med. Chem. 9, 687–695. 10.2174/138955709788452757 19519494

[B39] Luca V. Stringaro A. Colone M. Pini A. Mangoni M. L. (2013). Esculentin(1-21), an amphibian skin membrane-active peptide with potent activity on both planktonic and biofilm cells of the bacterial pathogen *Pseudomonas aeruginosa* . Cell. Mol. Life Sci. 70, 2773–2786. 10.1007/s00018-013-1291-7 23503622 PMC11113931

[B40] Luong H. X. Kim D. H. Lee B. J. Kim Y. W. (2018). Effects of lysine-to-arginine substitution on antimicrobial activity of cationic stapled heptapeptides. Arch. Pharm. Res. 41, 1092–1097. 10.1007/s12272-018-1084-5 30361948

[B41] Lyu Y. Chen T. Shang L. Yang Y. Li Z. Zhu J. (2019). Design of trp-rich dodecapeptides with broad-spectrum antimicrobial potency and membrane-disruptive mechanism. J. Med. Chem. 62, 6941–6957. 10.1021/acs.jmedchem.9b00288 31276398

[B42] Mangoni M. L. Shai Y. (2009). Temporins and their synergism against Gram-negative bacteria and in lipopolysaccharide detoxification. Biochim. Biophys. Acta - Biomembr. 1788, 1610–1619. 10.1016/j.bbamem.2009.04.021 19422786

[B43] Mangoni M. L. Epand R. F. Rosenfeld Y. Peleg A. Barra D. Epand R. M. (2008a). Lipopolysaccharide, a key molecule involved in the synergism between temporins in inhibiting bacterial growth and in endotoxin neutralization. J. Biol. Chem. 283, 22907–22917. 10.1074/jbc.m800495200 18550541

[B44] Mangoni M. L. Maisetta G. Di Luca M. Gaddi L. M. Esin S. Florio W. (2008b). Comparative analysis of the bactericidal activities of amphibian peptide analogues against multidrug-resistant nosocomial bacterial strains. Antimicrob. Agents Chemother. 52, 85–91. 10.1128/aac.00796-07 17954700 PMC2223874

[B45] Mangoni M. L. Mcdermott A. M. Zasloff M. (2016). Antimicrobial peptides and wound healing: biological and therapeutic considerations. Exp. Dermatol. 25, 167–173. 10.1111/exd.12929 26738772 PMC4789108

[B46] Miyazawa K. Mantel C. Lu L. Morrison D. C. Broxmeyer H. E. (1991). Lactoferrin-lipopolysaccharide interactions. Effect on lactoferrin binding to monocyte/macrophage-differentiated HL-60 cells. J. Immunol. 146, 723–729. 1702815

[B47] Morrison D. C. Ryan J. L. (1987). Endotoxins and disease mechanisms. Annu. Rev. Med. 38, 417–432. 10.1146/annurev.me.38.020187.002221 3555304

[B48] Nagaoka I. Tamura H. Reich J. (2020). Therapeutic potential of cathelicidin peptide LL-37, an antimicrobial agent, in a murine sepsis model. Int. J. Mol. Sci. 21, 5973. 10.3390/ijms21175973 32825174 PMC7503894

[B49] Nan Y. H. Park K. H. Park Y. Jeon Y. J. Kim Y. Park I. S. (2009). Investigating the effects of positive charge and hydrophobicity on the cell selectivity, mechanism of action and anti-inflammatory activity of a Trp-rich antimicrobial peptide indolicidin. FEMS Microbiol. Lett. 292, 134–140. 10.1111/j.1574-6968.2008.01484.x 19191872

[B50] Nan Y. H. Lee S. H. Kim H. J. Shin S. Y. (2010). Mammalian cell toxicity and candidacidal mechanism of Arg- or Lys-containing Trp-rich model antimicrobial peptides and their d-enantiomeric peptides. Peptides 31, 1826–1831. 10.1016/j.peptides.2010.07.003 20621141

[B51] Nan Y. H. Park I. S. Hahm K. S. Shin S. Y. (2011). Antimicrobial activity, bactericidal mechanism and LPS-neutralizing activity of the cell-penetrating peptide pVEC and its analogs. J. Pept. Sci. 17, 812–817. 10.1002/psc.1408 21956793

[B52] Oren Z. Ramesh J. Avrahami D. Suryaprakash N. Shai Y. Jelinek R. (2002). Structures and mode of membrane interaction of a short alpha helical lytic peptide and its diastereomer determined by NMR, FTIR, and fluorescence spectroscopy. Eur. J. Biochem. 269, 3869–3880. 10.1046/j.1432-1033.2002.03080.x 12180963

[B53] Rajasekaran G. Kamalakannan R. Shin S. Y. (2015). Enhancement of the anti-inflammatory activity of temporin-1Tl-derived antimicrobial peptides by tryptophan, arginine and lysine substitutions. J. Pept. Sci. 21, 779–785. 10.1002/psc.2807 26311041

[B54] Ramamourthy G. Park J. Seo C. Vogel H. J. Park Y. (2020). Antifungal and antibiofilm activities and the mechanism of action of repeating lysine-tryptophan peptides against Candida albicans. Microorganisms 8, 758. 10.3390/microorganisms8050758 32443520 PMC7285485

[B55] Rivas-Santiago B. Jacobo-Delgado Y. Rodriguez-Carlos A. (2021). Are host defense peptides and their derivatives ready to be part of the treatment of the next coronavirus pandemic? Arch. Immunol. Ther. Exp. Warsz. 69, 25. 10.1007/s00005-021-00630-9 34529143 PMC8444179

[B56] Rosenfeld Y. Lev N. Shai Y. (2010). Effect of the hydrophobicity to net positive charge ratio on antibacterial and anti-endotoxin activities of structurally similar antimicrobial peptides. Biochemistry 49, 853–861. 10.1021/bi900724x 20058937

[B57] Schmitt P. Rosa R. D. Destoumieux-Garzón D. (2016). An intimate link between antimicrobial peptide sequence diversity and binding to essential components of bacterial membranes. Biochim. Biophys. Acta - Biomembr. 1858, 958–970. 10.1016/j.bbamem.2015.10.011 26498397

[B58] Shagaghi N. Palombo E. A. Clayton A. H. Bhave M. (2016). Archetypal tryptophan-rich antimicrobial peptides: properties and applications. World J. Microbiol. Biotechnol. 32, 31. 10.1007/s11274-015-1986-z 26748808

[B59] Sinha M. Kaushik S. Kaur P. Sharma S. Singh T. P. (2013). Antimicrobial lactoferrin peptides: the hidden players in the protective function of a multifunctional protein. Int. J. Pept. 2013, 1–12. 10.1155/2013/390230 PMC3608178 23554820

[B60] Smirnova M. P. Kolodkin N. I. Kolobov A. A. Afonin V. G. Afonina I. V. Stefanenko L. I. (2020). Indolicidin analogs with broad-spectrum antimicrobial activity and low hemolytic activity. Peptides 132, 170356. 10.1016/j.peptides.2020.170356 32593681

[B61] Tang M. Waring A. J. Lehrer R. I. Hong M. (2008). Effects of guanidinium-phosphate hydrogen bonding on the membrane-bound structure and activity of an arginine-rich membrane peptide from solid-state NMR spectroscopy. Angew. Chem. Int. Ed. 47, 3202–3205. 10.1002/anie.200705993 18338418

[B62] Terri M. Mancianti N. Trionfetti F. Casciaro B. De Turris V. Raponi G. (2022). Exposure to b-LED light while exerting antimicrobial activity on gram-negative and -positive bacteria promotes transient EMT-like changes and growth arrest in keratinocytes. Int. J. Mol. Sci. 23, 1896. 10.3390/ijms23031896 35163819 PMC8837184

[B63] Travis S. M. Anderson N. N. Forsyth W. R. Espiritu C. Conway B. D. Greenberg E. P. (2000). Bactericidal activity of mammalian cathelicidin-derived peptides. Infect. Immun. 68, 2748–2755. 10.1128/iai.68.5.2748-2755.2000 10768969 PMC97484

[B64] Vogel H. J. Schibli D. J. Jing W. Lohmeier-Vogel E. M. Epand R. F. Epand R. M. (2002). Towards a structure-function analysis of bovine lactoferricin and related tryptophan- and arginine-containing peptides. Biochem. Cell Biol. 80, 49–63. 10.1139/o01-213 11908643

[B65] Wei L. Yang J. He X. Mo G. Hong J. Yan X. (2013). Structure and function of a potent lipopolysaccharide-binding antimicrobial and anti-inflammatory peptide. J. Med. Chem. 56, 3546–3556. 10.1021/jm4004158 23594231

[B66] Whitfield C. Trent M. S. (2014). Biosynthesis and export of bacterial lipopolysaccharides. Annu. Rev. Biochem. 83, 99–128. 10.1146/annurev-biochem-060713-035600 24580642

[B67] Yang S. T. Shin S. Y. Lee C. W. Kim Y. C. Hahm K. S. Kim J. I. (2003). Selective cytotoxicity following Arg-to-Lys substitution in tritrpticin adopting a unique amphipathic turn structure. FEBS Lett. 540, 229–233. 10.1016/s0014-5793(03)00266-7 12681513

[B68] Zelezetsky I. Tossi A. (2006). Alpha-helical antimicrobial peptides--using a sequence template to guide structure-activity relationship studies. Biochim. Biophys. Acta - Biomembr. 1758, 1436–1449. 10.1016/j.bbamem.2006.03.021 16678118

[B69] Zhang Q. Y. Yan Z. B. Meng Y. M. Hong X. Y. Shao G. Ma J. J. (2021). Antimicrobial peptides: mechanism of action, activity and clinical potential. Mil. Med. Res. 8, 48. 10.1186/s40779-021-00343-2 34496967 PMC8425997

[B70] Zhu X. Ma Z. Wang J. Chou S. Shan A. (2014). Importance of tryptophan in transforming an amphipathic peptide into a Pseudomonas aeruginosa-targeted antimicrobial peptide. PLoS One 9, e114605. 10.1371/journal.pone.0114605 25494332 PMC4262413

