# Supplementary material for: KDEON WK-11: A short antipseudomonal peptide with promising potential
Source: Front Chem. 2022 Nov 17;10:1000765. doi: 10.3389/fchem.2022.1000765 (PMC9713011; doi:10.3389/fchem.2022.1000765)
Supplement: Supplementary file 1 [file DataSheet1.docx]

**KDEON WK-11: A short antipseudomonal peptide with promising potential**

**Bruno Casciaro^1#^, Maria Rosa Loffredo^1#^, Floriana Cappiello^1^, Niamh O’Sullivan^1^, Carola Tortora^2^, Rizwan Manzer^3^, Sougata Karmakar^3^, Alan Haskell^3^, Syed K. Hasan^3^*, Maria Luisa Mangoni^1^**

^1^ Laboratory affiliated to Pasteur Italia-Fondazione Cenci Bolognetti, Department of Biochemical Sciences A. Rossi Fanelli, Sapienza University of Rome, Rome, Italy; ^2^ Department of Chemistry and Technology of Drugs, “Department of Excellence 2018–2022”, Sapienza University of Rome, ^3^ Iuventis Technologies Inc. (DBA Immunotrex Biologics), Lowell, MA, USA

**^*^Correspondence:**Syed K. Hasan. Email: shasan@immunotrex.com

# The two authors equally contributed to the work

**Keywords:** antimicrobial peptides, lipopolysaccharides, *Pseudomonas aeruginosa*, tryptophan, antibiofilm activity

Supplementary Material


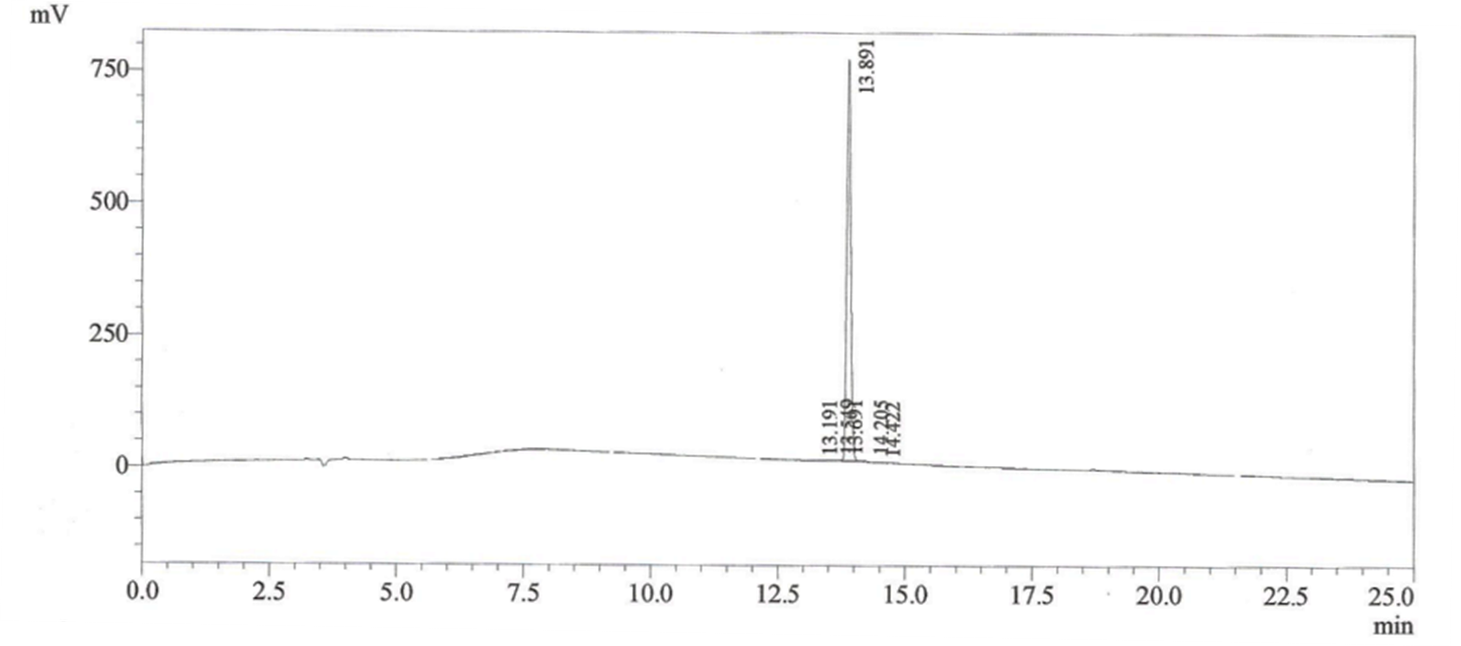


**Supplementary Figure 1.** High performance liquid chromatography (HPLC) spectrum of KDEON WK-11.
